# Editorial: Preeclampsia and the brain: Pre-clinical and clinical studies of cerebral involvement in preeclampsia

**DOI:** 10.3389/fphys.2023.1151091

**Published:** 2023-02-15

**Authors:** Lina Bergman, Carlos Alonso Escudero, Catherine Cluver, Roxanne Hastie, Pablo Torres-Vergara

**Affiliations:** ^1^ Department of Obstetrics and Gynecology, Institute of Clinical Sciences, Sahlgrenska Academy, University of Gothenburg, Gothenburg, Sweden; ^2^ Department of Obstetrics and Gynecology, Sahlgrenska University Hospital, Gothenburg, Sweden; ^3^ Department of Women’s and Children’s Health, Uppsala University, Uppsala, Sweden; ^4^ Department of Obstetrics and Gynecology, Stellenbosch University, Stellenbosch, South Africa; ^5^ Vascular Physiology Laboratory, Department of Basic Sciences, Universidad del Bío-Bío, Chillán, Chile; ^6^ Group of Research and Innovation in Vascular Health, GRIVAS Health, Chillan, Chile; ^7^ Mercy Perinatal, Department of Obstetrics and Gynaeology, Mercy Hospital for Women, Melbourne, VIC, Australia; ^8^ Department of Obstetrics and Gynaecology, Melbourne University, Melbourne, VIC, Australia; ^9^ Departamento de Farmacia, Facultad de Farmacia, Universidad de Concepcion, Concepcion, Chile

**Keywords:** preeclampsia, blood brain, barrier, neuroinflammation, cognition, pre-clinical, clinical

Preeclampsia was first recognized as a pregnancy related condition centuries ago when women presented with cerebral complications like eclampsia. Today, eclampsia is rarely seen in high-income settings, most likely because of improved antenatal care. Preeclampsia is often diagnosed earlier in these settings and timely delivery, magnesium sulphate and blood pressure control all play a role in the decreased incidence (Chappell et al., 2021). Unfortunately, in low-to-middle-income countries this is not the case (Duley, 2009).

Cerebral complications are one of the most common causes of maternal death. Despite this, there is a paucity of research focused on the brain and preeclampsia when compared to other organs like the cardiovascular system. Recently, large register-based studies have shown that women with preeclampsia, even without eclampsia, are at increased risks of neurological disorders. These disorders include dementia, epilepsy and cognitive decline which may occur months to years after the affected pregnancy (Poon et al., 2023). Imaging studies have shown cerebral edema in up to 20% of cases of preeclampsia (Fisher et al., 2016; Fang et al., 2017). Pre-clinical and clinical studies have demonstrated blood-brain barrier impairment, cerebral blood flow alterations and increased neuroinflammatory activity but how these findings correlate to long-term neurological complications are not entirely clear (Bergman et al., 2020; Bergman et al., 2021a; Bergman et al., 2021b; Escudero et al., 2022).

There have also been reports of poorer neurological outcomes in the offspring of women with preeclampsia. These include an increased risk of cerebral palsy, stroke, developmental delays, poor cognitive development, intellectual disability, anxiety, depressive symptoms, attention-deficit disorder, and hyperactivity. The causal link between preeclampsia and these outcomes are also poorly understood (Escudero et al., 2022).

In this Research Topic, we aimed to include articles highlighting the importance of cerebral involvement in preeclampsia for mothers and their children. We have included preclinical studies with animal models of preeclampsia assessing potential pathophysiological pathways, studies of brain imaging, the analysis of potential new diagnostic biomarkers of endothelial dysfunction and a mini-review.

Two studies describe potential pathophysiological pathways to cognitive impairment following preeclampsia. Johnson et al. show that in an experimental preeclampsia rat model, persistent endothelial and smooth muscle vascular dysfunction may contribute to hippocampal dependent impairment in long-term memory. Muhammad-Jones et al. report using a preeclampsia mouse model that there is decreased neuroinflammatory response in the hippocampus. This may in be important for later hippocampal and memory function.

McBride et al. show a potential link between impaired cardiovascular function and vascular dementia. Using brain imagining they found an increase in white matter lesions, which are associated with vascular dementia, among with impaired cardiovascular function tests. Interestingly, and in contrast to previous reports, these white matter lesions were not more common in women with a history of preeclampsia.

Carlberg et al. assessed the potential of endothelial biomarkers to identify women at risk of neurological complications and other end-organ complications. They showed that thrombomodulin may be a potential biomarker to identify those at greater risk.

Whitaker et al. found impaired growth and remodeling of cerebral vessels in offspring of a preeclampsia rat model. These findings were present in both juvenile and adult offspring. This may help explain the findings of poorer neurological outcomes in children born to women with preeclampsia from register studies.

Finally, a review by Korzeniewski et al. highlighted the need for large clinical studies with long-term follow up of the child in order to implement interventions to improve long-term health for children born to women with preeclampsia.

This Research Topic aimed to serve as a platform for researchers to publish novel findings in the field of cerebral complications of preeclampsia. We hope that these articles contribute to the current understanding of cerebrovascular alterations in women with preeclampsia and their offspring and that in the near future, new strategies are developed to follow up women who have had preeclampsia and their children.
